# Engineering calcium signaling of astrocytes for neural–molecular computing logic gates

**DOI:** 10.1038/s41598-020-79891-x

**Published:** 2021-01-12

**Authors:** Michael Taynnan Barros, Phuong Doan, Meenakshisundaram Kandhavelu, Brendan Jennings, Sasitharan Balasubramaniam

**Affiliations:** 1 School of Computer Science and Electronic Engineering, University of Essex, Colchester, UK; 2 BioMediTech, Faculty of Medicine and Health Technology, Tampere University, P.O.Box 553, 33101 Tampere, Finland; 3 Telecommunication Software and Systems Group (TSSG), Waterford Institute of Technology (WIT), Waterford, Ireland; 4 FutureNeuro, The SFI Research Centre for Chronic and Rare Neurological Diseases, RCSI University of Medicine and Health Sciences, Dublin, Ireland

**Keywords:** Biomedical engineering, Computer science, Biotechnology, Computational biology and bioinformatics

## Abstract

This paper proposes the use of astrocytes to realize Boolean logic gates, through manipulation of the threshold of $\hbox {Ca}^{2+}$ ion flows between the cells based on the input signals. Through wet-lab experiments that engineer the astrocytes cells with pcDNA3.1-hGPR17 genes as well as chemical compounds, we show that both AND and OR gates can be implemented by controlling $\hbox {Ca}^{2+}$ signals that flow through the population. A reinforced learning platform is also presented in the paper to optimize the $\hbox {Ca}^{2+}$ activated level and time slot of input signals $T_b$ into the gate. This design platform caters for any size and connectivity of the cell population, by taking into consideration the delay and noise produced from the signalling between the cells. To validate the effectiveness of the reinforced learning platform, a $\hbox {Ca}^{2+}$ signalling simulator was used to simulate the signalling between the astrocyte cells. The results from the simulation show that an optimum value for both the $\hbox {Ca}^{2+}$ activated level and time slot of input signals $T_b$ is required to achieve up to 90% accuracy for both the AND and OR gates. Our method can be used as the basis for future Neural–Molecular Computing chips, constructed from engineered astrocyte cells, which can form the basis for a new generation of brain implants.

## Introduction

Synthetic biology has facilitated capabilities to engineer biological cells that can lead to novel applications in medicine as well as in biotechnology^1–4^. This engineering process is realized through the synthesis of genetic circuits that results in new cell functions; an example is controlling cellular intra and inter-communications. Numerous digital-like devices have emerged from synthetic biology, including toggle switches^5^, oscillators^6^, as well as Boolean logic gates^7^. Boolean logic gates, in particular, have received considerable attention due to their ability to be assembled into logic circuits that can perform computation, which can be used to reconfigure cellular operations for therapeutic purposes, or diagnostic sensing of multiple enzymes that are indicators of diseases^8–10^. Examples of Boolean logic gates that have been engineered from cells includes AND^11^, OR^12^, NOR^13^, and XOR^14^ gates. An essential function in synthetic logic circuits is communication, and this can be short-range between different genes within a circuit, to communication between populations of cells that represent individual gates. Therefore, engineering molecular communication between the cells through engineered genetic circuits can not only produce logic gates with multiple computational functions, but can enable reconfigurability of the logic operations^15–18^. Molecular communication is an emerging paradigm that aims to characterize as well as engineer biological communication systems using communication engineering theory in conjunction with synthetic biology^19–22^. Synthetic circuits to control molecular communications use ligand-responsive transgene systems that can respond to a particular stimulus^12,23,24^; this can enable reconfiguration when specific signalling molecules activate the circuit.

**Figure 1:**
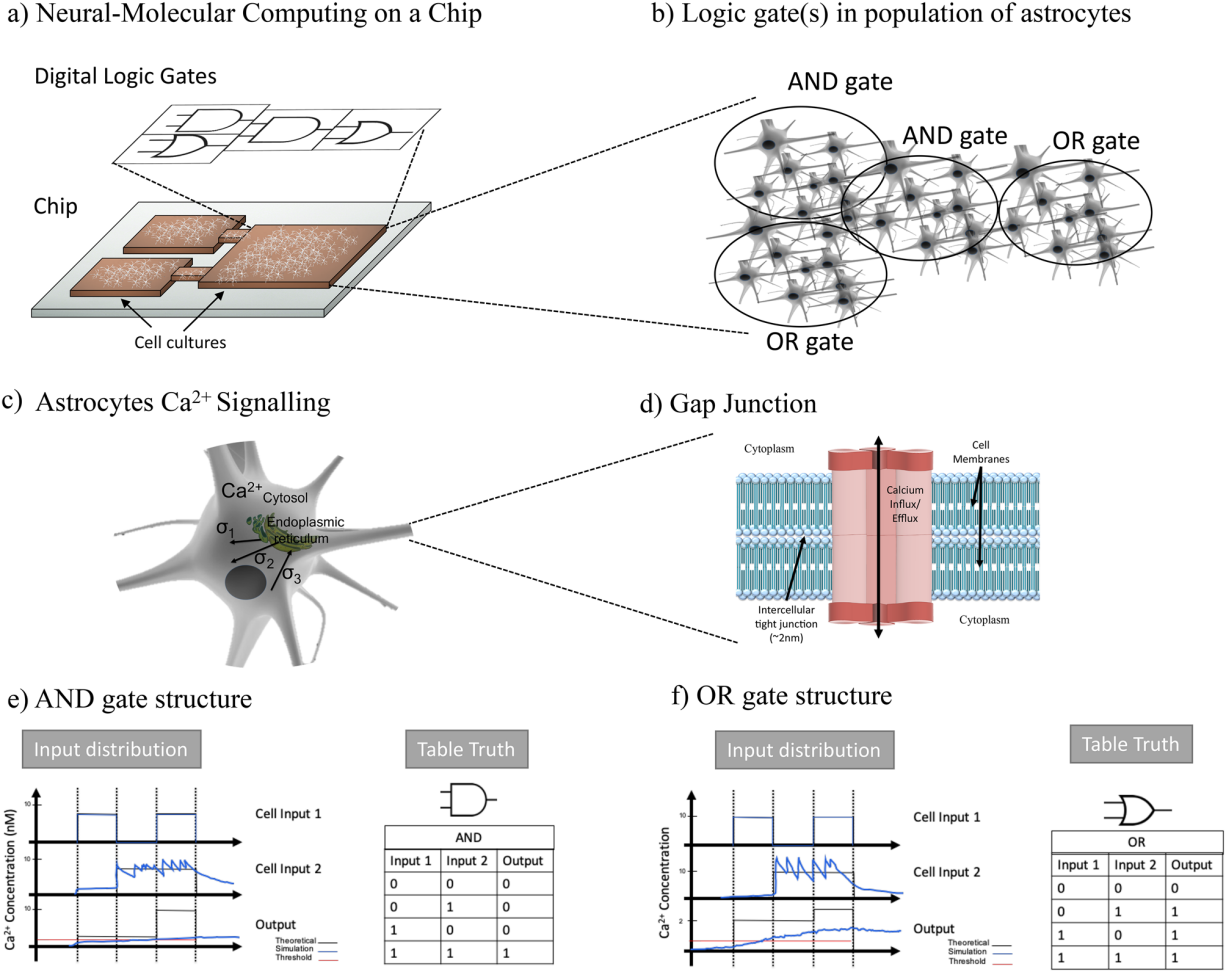
Neural–molecular computing on a neural–molecular computing chip (**a**), is composed of digital logic gates that are implemented from engineered astrocytes cells (**b**). The inputs, 1 and 2, to the astrocyte cells are the incoming $\hbox {Ca}^{2+}$ signals into the gate, which is computed to produce an output signal. The digital gate behaviours are achieved by engineering the threshold of the intracellular $\hbox {Ca}^{2+}$ signalling process (**c**) and the gap junctions that facilitate cell-cell $\hbox {Ca}^{2+}$ diffusion (**d**). Simulation illustration of $\hbox {Ca}^{2+}$ signals through cells that represent an AND gate (**e**) as well as an OR gate (**f**).

In this paper, we attempt to create Boolean logic gates from eukaryotic cells, namely astrocytes (Fig. 1a–f). Astrocytes are glial cells that are found in brain tissue and have a particular function in supporting stability of the neurons. This stability comes in the form of structural support for neurons, providing nutrients and oxygen supply, pathogen destruction, and removal of dead cells. To create logic gates from astrocytes (Fig. 1e,f), our approach is based on engineering the $\hbox {Ca}^{2+}$ signalling (Fig. 1c,d) between the cells. This is achieved by using synthetic genes that will set the threshold value for the sensitivity of the intracellular $\hbox {Ca}^{2+}$ signalling. $\hbox {Ca}^{2+}$ signalling is a short-range inter-cellular communication process that uses ions as the signalling molecules between the gap junction connection of the cells. In our previous work^25,26^, an engineered $\hbox {Ca}^{2+}$ signalling-based molecular communication system was analyzed to understand the short-range communication properties, in particular the behaviour of ion propagation throughput within a tissue. The logic gate structure depicted in Fig. 1a, includes three populations of cells that each corresponds to a link with a logic gate (two input links and one output link). The two input links of $\hbox {Ca}^{2+}$ signalling populations represent the input into the digital logic gates, which are then transmitted to the output link population with the engineered threshold to perform the logic gate operation. The threshold control of the output link population will determine if it operates as either an AND or an OR gate. The logic circuits built from astrocytes could provide the basis for a new breed of Neural–Molecular Computing on a Chip, as illustrated in Fig. 1a,b, which could transform implantable brain chips which to date have predominately been developed from non-biological materials. However, to fully realize logic circuits built from astrocytes and rely on $\hbox {Ca}^{2+}$ signalling as part of its operation, there are many challenges arising from the stochastic behaviour of the signalling nature of the cell; this includes:

1. *Impact from self-regulating spontaneous signalling of* $\hbox {Ca}^{2+}$ *ions:* The stochastic nature of $\hbox {Ca}^{2+}$ signalling leads to unpredictable stimulation and propagation of $\hbox {Ca}^{2+}$ ions that result in noise that can affect the reliability of the logic operation.
2. *Inter-cellular* $\hbox {Ca}^{2+}$ *propagation delay:* Inter-cellular signalling is prolonged compared to conventional CMOS bus lines found in digital logic gates. This property can lead to issues in synchronizing the communication between the cells during the logic operation. Therefore, modelling and characterizing the astrocyte network is critical to ensure the optimal population of astrocytes that will minimize false positive or negative results from the logic gate operation.
3. *Impact from uncertainty in the astrocyte cells network structure:* The unknown network topology of the astrocyte cells can lead to different delays that can impact the logic operation reliability.

To address these challenges, we have developed a reinforced-learning platform that is used to assist the design of the logic gate from the astrocyte cells. The reinforced-learning algorithm analyses the molecular communication of $\hbox {Ca}^{2+}$ ions within the astrocyte network, and through a state value function learning process, adapts parameters that determine the threshold level needed to be engineered in the cells to encode active states of $\hbox {Ca}^{2+}$ ions in the output link. Figure 1e,f depict the gate function for a small population of astrocytes, where we show through theoretical simulations the processing of $\hbox {Ca}^{2+}$ signals in the output link population, and compared it to the truth table of the respective logic gate. We summarise the paper’s contribution as follows:

- **Eukaryote cell-based synthetic logic gate:** In contrast to previous approaches that use prokaryotic cells for developing the gates, we propose a model for developing AND and OR logic gates from astrocyte cells.
- **In-vitro experimentation:** Experiments performed using hGPR-17 synthetic gene expression in astrocytes as well as two sets of chemical compounds (*MDL*29, 951 and *T*0510.3657) that are used to elevate the $\hbox {Ca}^{2+}$ ion concentration and differentiate between the two types of gates. The experiment conducted in a petri dish demonstrates an AND and OR gate operations based on threshold control of the $\hbox {Ca}^{2+}$ signalling.
- **Reinforced learning platform for logic gates design:** The reinforced learning algorithm uses a closed-loop feedback system to fine-tune the astrocyte cell-cell communication parameters including the activation of $\hbox {Ca}^{2+}$ threshold and communication period, that will ensure the engineered population of logic gate cells can be integrated into a tissue and operate reliably. This will enable the platform to be used for future practical applications that require engineered astrocytes to perform logic operations. The adaptive process tunes the $\hbox {Ca}^{2+}$ signalling activated level to determine the optimal control of ions flow that will result in reliable gate operation.
- **Accuracy and delay analysis:** Due to the fluctuation behaviour of cell-cell communication with inter-cellular $\hbox {Ca}^{2+}$ signalling, we theoretically analyze the accuracy of the logic operation, as well as the delay of input flow to the logic gate using *static timing analysis* from conventional digital logic circuit theory.

## Methodology

**Figure 2:**
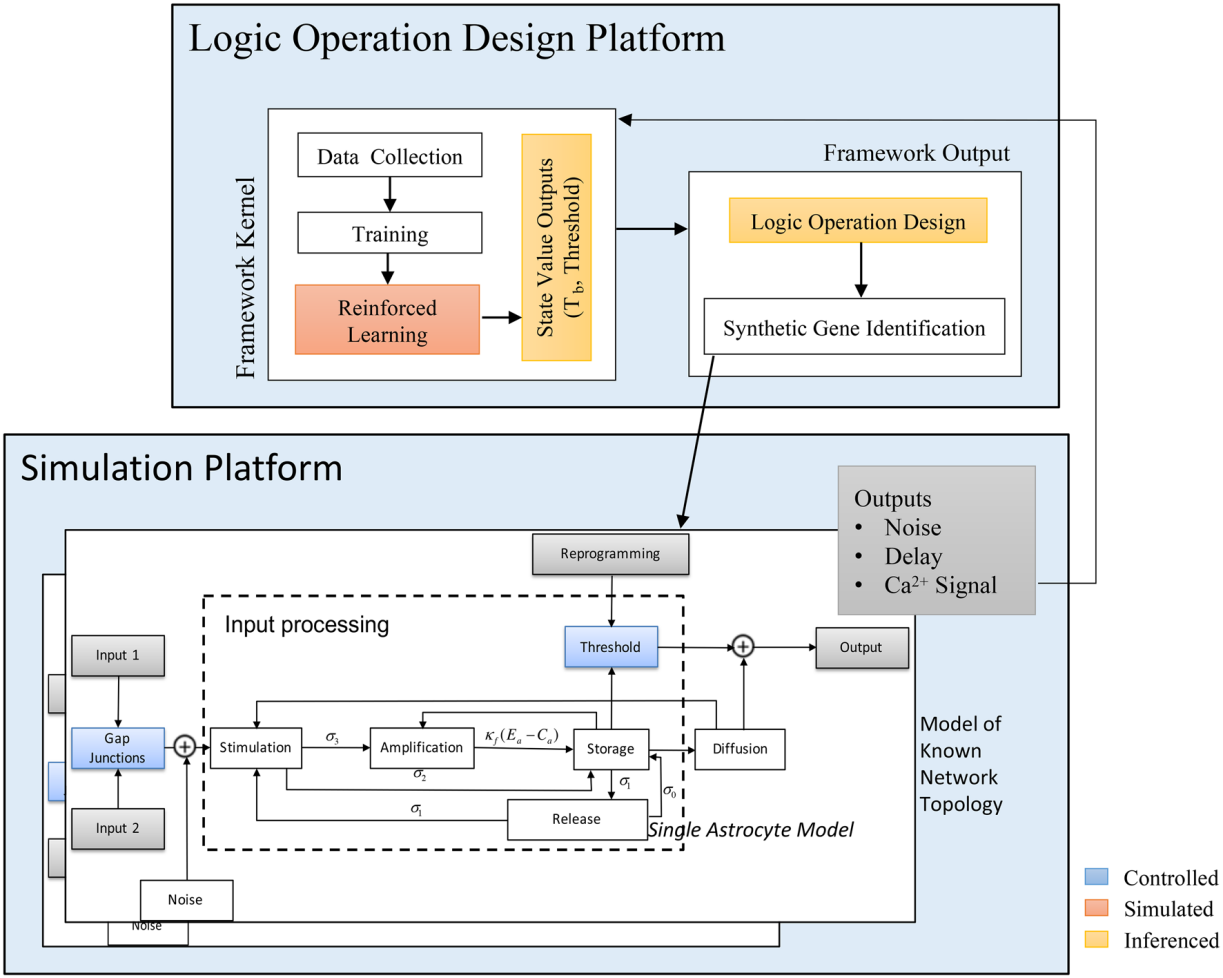
Astrocyte-based logic gate design platform using reinforced learning. The platform is a feedback system for reinforced learning using state value functions that fine tunes the $\hbox {Ca}^{2+}$ activated level as well as time slot for input signals $T_b$. The Kernel of the platform considers information such as the delay of signals between cells in the population, noise, transmission period, required logic operation, input signal flow concentration of $\hbox {Ca}^{2+}$ ions, which are fed into the data collection that is used for training. In the output of the platform, synthetic gene transcription is identified for the logic operation based on the defined values of the cellular signalling threshold the is tuned by the kernel state value functions. To validate the platform, a $\hbox {Ca}^{2+}$ signalling-based molecular communications simulator is integrated. The simulator includes models for individual cell’s intra as well as intercellular signalling for a defined topology. The output population will produce $\hbox {Ca}^{2+}$ signals based on the logic computation. In blue are the controlling blocks that define the threshold for the logic gate operation. The blocks within the ”Input Processing” are stages of the $\hbox {Ca}^{2+}$ signalling process in each astrocyte cell.

Our methodology involves both wet-lab experiments as well as theoretical simulations of the astrocyte-based logic gates. Engineering logic operations in a network of astrocyte cells must consider the cell-cell $\hbox {Ca}^{2+}$-signalling and its impact on the reliability of the computing functioning. This includes considering their internal signalling pathways and its relation to the engineered threshold, gap junctions probabilities for $\hbox {Ca}^{2+}$ ion propagation, as well as the delay of signals, which is dependent on the network connectivity. The random connectivity of the astrocyte cells network will lead to varying noise, delay and signal fading, which impacts on the reliability performance of the logic gates. In order to ensure a high reliability of the logic operation, we use a Reinforced Learning platform illustrated in Fig. 2, which will take as input the culture of astrocyte cells and based on end-to-end $\hbox {Ca}^{2+}$ signaling through the culture, will fine tune two parameters which are the optimal $\hbox {Ca}^{2+}$ activated level to be engineered into the cells and the optimal transmission period ($T_b$). In this paper, we used a $\hbox {Ca}^{2+}$ Signaling-based Molecular Communications simulator (bottom layer of Fig. 2) to simulate the astrocyte culture. The reinforced learning platform utilizes functions, which are designed to adapt system state variables of the inputs by recursively averaging the configuration parameters, and this will result in the optimal $\hbox {Ca}^{2+}$ activated level and the transmission period ($T_b$).

This section will present the Reinforced Learning platform that includes the $\hbox {Ca}^{2+}$ Signalling-based Molecular Communications simulator, as well as the methodology for the wet lab experiments.

### Synthetic logic gate design platform using reinforced learning

#### Reinforced Learning Platform

The core parts of our proposed platform illustrated in Fig. 2 are the Kernel and the Output. While the Kernel is responsible for setting the learning rules from the state value functions, the output implements the learning rules that set the $\hbox {Ca}^{2+}$ signalling activated level values for the AND or the OR gates. The framework converges the $\hbox {Ca}^{2+}$ signalling activated level as well as $T_b$ values based on input data of the astrocyte network to the Kernel (while this will come from the culture directly, in our case we are using the simulator to produce the input data). The Kernel is the more complex part, whereby the processed cellular population input data undergoes the training and the reinforced learning process. For simplicity, we used the cellular population input values of the framework as training features directly. As illustrated in Fig. 2 the simulator of the $\hbox {Ca}^{2+}$ signalling molecular communications in the astrocytes population will refine and converge the values of the optimal $\hbox {Ca}^{2+}$ activated level and $T_b$, by minimizing the noise and delay as signals are transmitted through the population, which makes the design agnostic to any network topology of astrocytes. Once the optimal $\hbox {Ca}^{2+}$ activated level and $T_b$ values are identified for the specific astrocyte population, a synthetic circuit is designed to stimulate $\hbox {Ca}^{2+}$ signal in the output link once the flow of ions from the two input links reaches the threshold.

#### $\hbox {Ca}^{2+}$ signalling-based molecular communications model

The astrocyte cell communication is characterized by both the intracellular as well as the intercellular signalling processes, and the simulator for this signaling process, shown in the bottom layer of Fig. 2), is described as follows: The intracellular $\hbox {Ca}^{2+}$ signalling (Fig. 1c,d) is based on the classical Goldbeter et al. model^27^. The model is based on stimulating the $\hbox {IP}_3$ protein, resulting in $\hbox {Ca}^{2+}$ signals generation and release (*Stimulation*). The released $\hbox {IP}_3$ indirectly controls the influx of $\hbox {Ca}^{2+}$ ions to the endoplasmic reticulum and its storage in the cytosolic area (*Storage*). Besides the stimulation process, certain cellular components, such as the endoplasmic reticulum and mitochondria, are also capable of self-generating $\hbox {Ca}^{2+}$ ions (*Amplification*). Finally, the exchange of $\hbox {Ca}^{2+}$ ions is conducted in two ways: cell-cell communication (*Diffusion*) and aleatory exchange of $\hbox {Ca}^{2+}$ to the extracellular space (*Release*). In inter-cellular $\hbox {Ca}^{2+}$ signalling, ions are propagated through the cellular tissues via a physical gate that connects the cytosolic areas of two neighbouring cells, and these gates are called *Gap Junctions*. Figure 1d shows how the gap junctions connect two cytosols. The gap junctions are composed of two *connexons*, one in each connecting cell, which is formed by six proteins called *connexins*. Inter-cellular diffusion only occurs when both connexons open at the same time. The voltage-sensitive gap junctions are assumed to have two states of conductance for each connexin: an open state with high conductance and a closed state with low conductance. For astrocytes, the gap junctions are closed around 18% of the time on average, which probabilistically dictates the cell-cell propagation of $\hbox {Ca}^{2+}$ ions that can subsequently interfere with the logic gate operation^26^. The theoretical modelling of the inter/intra $\hbox {Ca}^{2+}$ signalling with the gap junction diffusion of astrocytes population is presented in the Supplementary Material.

### Wet-lab experimental set-up

The in-vitro experiments aims to determine the sensitivity of the astrocyte cell culture to the induced $\hbox {Ca}^{2+}$ signals. This will determine the validity of the engineered threshold that differentiates between the AND and OR gates. Specifically, we targeted a population of astrocyte cells that have their thresholds controlled by the hGPR-17 gene and $\hbox {Ca}^{2+}$ signals induced by either the MDL29,951 or T0510.3657 chemical compounds. The two gates were programmed to induce fluorescent light with increasing $\hbox {Ca}^{2+}$ concentration values as the concentration of ions crosses the threshold to indicate a successful logic operation. The $\hbox {Ca}^{2+}$ signal output of one astrocyte population is the input to a neighbouring population, and this intercellular signalling will be defined by threshold value from the reinforced learning platform. This intercellular signalling activation process can be further explored in future works by having a unique threshold setup for different cell types. Our experimental design is based on the approach in^28^.

#### Cell culture and hGPR-17 gene expression

Human astrocytoma cells, 1321N1, were cultured in Dulbecco’s modified Eagle’s medium with L-glutamine (DMEM-high glucose) (Sigma-Aldrich) supplemented with 10% (v/v) fetal bovine serum (FBS) (Sigma-Aldrich), penicillin and streptomycin (100U/ml) (Sigma-Aldrich), sodium pyruvate 1mM (Sigma-Aldrich), and amphotericin B 250 $\upmu$g/ml (Sigma-Aldrich) and grown at 37${^\circ }$C in $\hbox {CO}_2$ incubator. Cells were seeded in a 25 cm$^{2}$, T-25 flask (Fennokauppa) and after 24 h of incubation, hGPR17 plasmid was transfected with $\hbox {Ca}^{2+}$ phosphate transfection kit (Sigma-Aldrich). The hGPR17 gene was cloned into pcDNA3.1 plasmid, which is a mammalian expression vector^29^. 3 $\upmu$g of pcDNA3.1-hGPR17 plasmid was used to transfect 1321N1. Post transfection of the plasmid, the media was removed, and fresh DMEM containing 10% FBS was replaced. For the $\hbox {Ca}^{2+}$ time-lapse analysis MMK1 cells, GBM cells derived from patient samples which overexpressed GPR17 were plated in 96-well plates at an initial density of 1104 cells per well. The cells were incubated overnight to reach around 70% of confluence. To measure $\hbox {Ca}^{2+}$ level change over time, the cells were incubated with 5 $\upmu$M Fura-2 AM (Sigma-Aldrich, St. Louis, MO, USA) for 30 min at 37${^\circ }$C. The cells were washed with PBS twice before adding 50 $\upmu$L complete medium. Then, 50 $\upmu$L of PBS containing 25 $\upmu$M concentration of the MDL29,951 or T0510.3657 were added to the wells, and $\hbox {Ca}^{2+}$ changes were measured immediately for 1.5 h. For testing the signalling communication, we have used the cell line model, 1321N1 which does not express GPR17 or other P2Y like receptors and Uracil nucleotide receptors. Upon transfection, the cell line over-expresses the GPR17 and hence addition of ATP or MDL29,951 or T 0510.3657 specifically binds and activates GPR17 receptor and affects intracellular calcium signalling.

#### Quantification of cellular $\hbox {Ca}^{2+}$ signals

The level of cellular $\hbox {Ca}^{2+}$ was quantified using the Fura2-AM Assay kit (Sigma-Aldrich). 24 h of post-transfection, transient cell line was collected on 96-well plates at a concentration of 1 $\times 10^5$ cells/well. Cells were incubated with increasing concentrations of signalling molecules, MDL 29951 (Abcam), and T0510.3657 (AKos Consulting & Solutions Deutschland GmbH) at 37${^\circ }$C for 2 h. 10 $\upmu$M Fura 2-AM was added to the cells and then assayed for $\hbox {Ca}^{2+}$ accumulation after the 30 minutes of incubation at 37${^\circ }$C, following the manufacturer’s instructions. The difference between the fluorescence level of the control and signalling molecule treated samples were measured using the plate reader (Ascent). In the experimental set up following conditions were used: (1) Cells without the transfection of plasmid, (2) Cells with the transfection of plasmid and without the compound incubation, (3) Cells without the transfection of plasmid and with the incubation of 50 $\upmu$M concentration of compounds, (4.a) Cells with the transfection of plasmid and with the incubation of 25 $\upmu$M of compound and (4.b) 50 $\upmu$M of the concentration of compounds. To quantify the changes in $\hbox {Ca}^{2+}$ level, the kit protocol, as given by the vendor, was used. Technical and biological repeats were used to measure fluorescence and were averaged. The fluorescent signal was measured using a microplate reader (Spark$\circledR$, Tecan) at two dual excitation/emission wavelengths of 340/510 nm and 380/510 nm.

## Results

### Wet lab experiments

**Figure 3:**
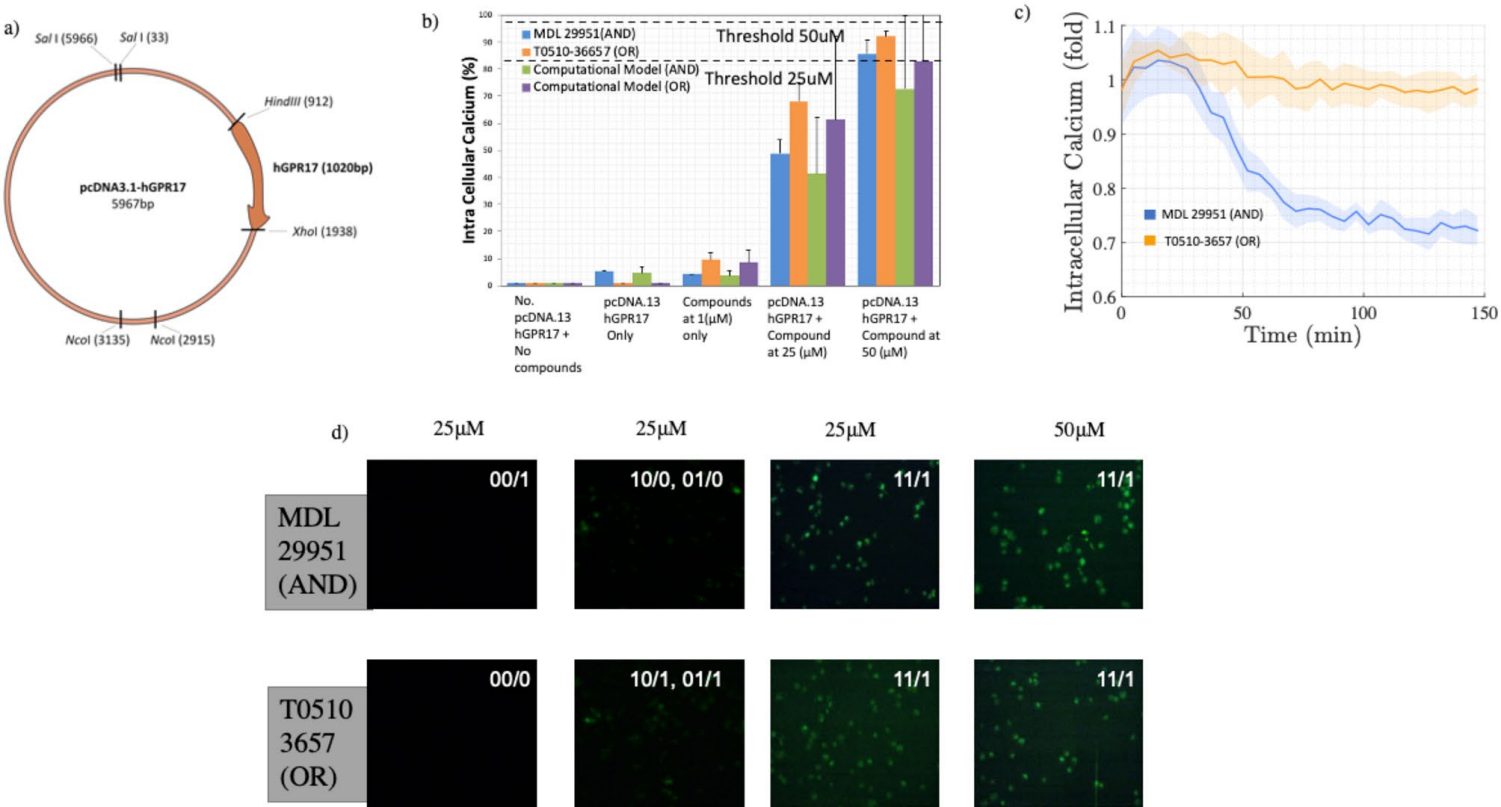
Wet lab experiments of the logic gate input control for an astrocyte population in-vitro. (**a**) The pcDNA3.1-hGPR17 plasmid used to engineer the threshold of the astrocytes in the experiments. In (**b**) the percentage of intracellular $\hbox {Ca}^{2+}$ concentration over five in-vitro setups: no pcDNA3.1-hGPR17 (no gene), pcDNA3.1-hGPR17 (genes only), no pcDNA3.1-hGPR17 with compounds (compounds only), pcDNA3.1-hGPR17 with compounds at 25 $\upmu$M (genes and compounds) and pcDNA3.1-hGPR17 with compounds at 50 $\upmu$M (genes and compounds). We also have provided an comparison between the experimental results and the computational models for AND and OR gates. Thresholds values are set based on the gate type as well as the $\hbox {Ca}^{2+}$ signal levels coming from the gates inputs. Based on these results, we observe the levels of fluctuation of the $\hbox {Ca}^{2+}$ signals that affect the performance of the logic operations. In (**c**) we show the intracellular $\hbox {Ca}^{2+}$ variation over time for both compounds. In (**d**) we show the increasing $\hbox {Ca}^{2+}$ concentration observed by the fluorescent light effect of in-vitro astrocyte cultures with compound concentrations of 25 $\upmu$M and 50 $\upmu$M for the AND gate with the *MDL*29, 951 compound and the OR gate with the *T*0510.3657.

Figure 3 presents the wet lab experiments to demonstrate the AND and OR gates that are engineered from the astrocyte cells. Figure 3a illustrates the engineered plasmid with the gene pcDNA3.1-hGPR17 insertions that are used to amplify the $\hbox {Ca}^{2+}$ signals for the two gates. The OR gate is a combination of *T*0510.3657 compound added to the gene pcDNA3.1-hGPR17, while the AND gate is a combination of *MDL*29, 951 with the gene pcDNA3.1-hGPR17. The compounds are used to amplify the $\hbox {Ca}^{2+}$ signals in the population based on the level of $\hbox {Ca}^{2+}$ signals coming from natural astrocytes, which are the inputs of the gates. In the case of 25 $\upmu$M, the input $\hbox {Ca}^{2+}$ signals are found in high concentration as opposed to 50 $\upmu$M, where the input $\hbox {Ca}^{2+}$ signals are in low concentration.

In order to evaluate the effectiveness of the logic operation, Fig. 3b illustrates the different quantity of $\hbox {Ca}^{2+}$ produced with respect to the different amounts of compounds added: non-engineered cells with no compounds (no incoming $\hbox {Ca}^{2+}$ signal inputs), only engineered cells, and non-engineered cells with only compounds produces a small quantity of $\hbox {Ca}^{2+}$ signals. However, the combinations of engineered cells with compounds (both 25 and 50 $\upmu$M) presents the amplification of $\hbox {Ca}^{2+}$ signals based on the AND and OR gates’ inputs from natural astrocytes. The threshold determines if a 1 output will be produced depending on the type of gates as well as input. In the case of 25 $\upmu$M the AND gate will produce small elevated quantity of $\hbox {Ca}^{2+}$ signals that will require two input of $\hbox {Ca}^{2+}$ signals to reach a high enough threshold. However, in the case of the OR gate, the elevated $\hbox {Ca}^{2+}$ signals is quite high that it will only require 1 input of $\hbox {Ca}^{2+}$ signals to reach and surpass a specific threshold. However, in the case of 50 $\upmu$M we can observe that the quantity of $\hbox {Ca}^{2+}$ ions produced are very high. Therefore, this means that the gates with 50 $\upmu$M solutions will be used for situation where input $\hbox {Ca}^{2+}$ signals from natural cells are very low (the two gates can still be created, and will only require small signal concentration input to reach a threshold). In Fig. 3b we also show results for the computational model of AND and OR gates, it can be observed that the computational results approximate the experimental results, demonstrating the same overall behaviour. However, the computational models contain more results variability; this is due to the organisation of the topology in the simulations, as well as the lack of measurement of all the effects of the direct calcium amplifications through the combination of the plasmid and chemical compounds.

Figure 3c presents the changes in overall stability and fluctuations of the $\hbox {Ca}^{2+}$ signals with respect to time and shows that the AND gate will have a more stable production of the signals compared to an OR gate, which will result in signals that fade after a certain period. The fluctuation of $\hbox {Ca}^{2+}$ signals is found throughout all configurations and logic operations, producing an average variation of 2.7% of internal signalling capacity, with a peak at pcDNA3.1-hGPR17 with compounds of 25 $\upmu$M with 6% fluctuation for both compounds. The regulatory $\hbox {Ca}^{2+}$ intracellular mechanisms associated with the cell-cell communication can produce random fluctuations. Figure 3d presents the fluorescent output of the cells based on the input signals (xx/y refers to the x being the input and y being the output). The result shows that the 25 $\upmu$M produces a certain level of output, but this is lower than in the case of 50 $\upmu$M. After addition of a drug (MDL29,951 and T 0510.3657), the receptor signalling gets activated in 15 minutes and the continuous stimulation have been observed for more than 2 h in live cell. The logic gates were observed to perform as long as the receptor signalling is activated.

### Simulation experiments

**Figure 4:**
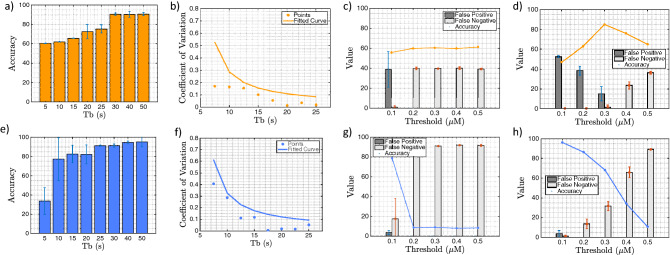
Accuracy analysis of a small astrocyte population with AND (top row) and OR (bottom rows) logic gate operation. The accuracy of the AND (**a**) and OR (**e**) logic gate operation over the pulse period $T_b$. Fluctuation levels of $\hbox {Ca}^{2+}$ versus the $T_b$ variation and the obtained regression model curve for AND (**b**) and OR (**f**) gates. Accuracy, false positive and false negative results with respect to varying the threshold values for low values of $T_b$ for AND (**c**) and OR (**g**) gates. Accuracy, false positive and false negative results with respect to varying the threshold values for high values of $T_b$ for AND (**d**) and OR (**h**) gates.

Figure 4 presents results on the logic computing simulation accuracy for the astrocyte cells AND as well as OR gates. The accuracy analysis of a small population of astrocytes AND logic gate is shown in the top row of Fig. 4. The aim of our analyses is to understand the impact that variation of $\hbox {Ca}^{2+}$ activated level and the $T_b$ of input signals will have on the gate’s computing reliability under low noise effects. Figure 4a shows directly increasing $T_b$ benefits the accuracy, giving peak performance around $90 \%$ for the AND logic operation. This is due to the impact of longer duration of $\hbox {Ca}^{2+}$ signals that can exist in the cell population, as shown in Fig. 3c, especially for AND gate, resulting in improved results when higher values of $T_b$ are used. $\hbox {Ca}^{2+}$ signalling fluctuations are represented as the statistical errors of the temporal series shown in Fig. 4b, which a simple regression curve shows have an inverse relationship with the $T_b$. Figure 4c shows the accuracy, false positive and false negative results for variations in the threshold values for a low level of $T_b$. As shown in the results, the accuracy is not affected by the threshold variation for low $T_b$, and stabilizes around 55–60%, whilst the false positive rate has an average of $8\%$ and the false negative rate an average of $16\%$. Low values of $T_b$ results in high fluctuation, as can be seen in Fig. 4b, which, even with different thresholds, does not affect the logic operation accuracy. This is contrary to Fig. 4c/d, which present the accuracy, false positive and false negative results for variations in the threshold values for high values of $T_b$. An optimal point is observed when the threshold value is around $30\%$ of the intracellular signalling capacity, with accuracy at $80\%$, false-positive at $15\%$ and false-negative at $2\%$. Higher thresholds values are likely due to the interference error in the output due to the increase in false negatives effects that are caused by $\hbox {Ca}^{2+}$ concentration fluctuations. The data from all the results presented in Fig. 4a–d that was input into the reinforced learning algorithm, resulted in the optimum value of $T_b = 30s$ and optimal $\hbox {Ca}^{2+}$ activated level of $0.3\, \upmu$M, which resulted in an accuracy of 90% with a minimum percentage of false positives and false negatives.

The accuracy analysis of a small population of astrocyte OR logic operation is presented in the bottom row of Fig. 4. The results show that higher levels of fluctuations in Fig. 4e,f, does not inhibit the OR logic operation from reaching $95\%$ of peak accuracy. However, the high level of fluctuations impacts on the results in Fig. 4g,h, where higher false negatives are found (average of $75\%$ for Fig. 4g and $41.4\%$ for Fig. 4h). At the same time, the increase in the threshold values appears to impact on the overall gate performance. Higher levels of fluctuations in low values of $T_b$s are responsible for this effect due to the increase in the false negatives that results from high values of $T_b$. When this data is input into the reinforced learning platform, the optimum value outputs are $T_b = 50s$, and optimal $\hbox {Ca}^{2+}$ activated level of $0.1\,\upmu$M, and this results in an accuracy of $98\%$ with a minimum percentage of false positives and negatives.

**Figure 5:**
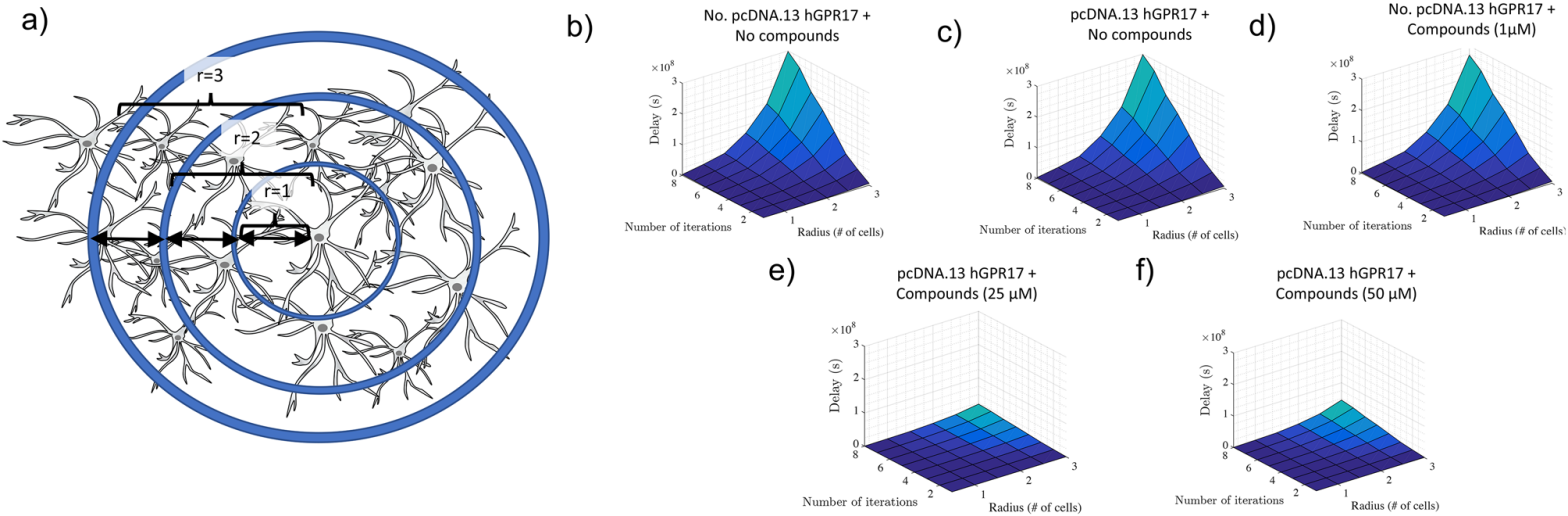
Static timing analysis for delay performance of the input population. (**a**) The static timing analysis of the input population with different reach radius *r*. (**b**) Delay in seconds for eight different values of *r* over 0 to 100 logical operations for no pcDNA3.1-hGPR17. (**c**) Delay results for pcDNA3.1-hGPR17 only. (**d**) Delay results for no pcDNA3.1-hGPR17 but with compounds. (**e**) Delay results for pcDNA3.1-hGPR17 with compounds at 25 $\upmu$M. (**f**) Delay results for pcDNA3.1-hGPR17 with compounds at 50 $\upmu$M.

Our simulations also include the static timing analysis to determine the $\hbox {Ca}^{2+}$ signal propagation delay through the astrocyte population and its impact on the logic gate operation. The simulation considers the data from the wet lab experiments. Figure 5a illustrates the topology of astrocytes population that is used for simulating the signal propagation through the output link. As shown in the figure, the topology is based on varying radius *r* of the astrocyte population for the input link. Figure 5b presents the delay results for the non-engineered cell and shows that as the number of operations increase, this also increases the delay with an increase in population radius *r*. Figure 5c presents the delay results for the cells that are engineered with the pcDNA3.1-hGPR17 genes with no compounds applied, while Fig. 5d presents the delay results for non-engineered cells but with the compounds applied. Figure 5e presents the delay results for the engineered cells with pcDNA3.1-hGPR17 applied with compounds at 25 $\upmu$M, and Fig. 5f with compounds at 50 $\upmu$M. The high delay as signals propagate through the population in Fig. 5b–d is due to the propagation of normal levels of $\hbox {Ca}^{2+}$ ions that are in each cells. However, in the case of Fig. 5e,f, the $\hbox {Ca}^{2+}$ ions are amplified, and this leads to a larger quantity of concentration that is pushed from cell to cell, resulting in a higher speed of propagation, leading to lower end-to-end delay. Based on fast increase response of many compounds, the results show that the delay has decreased by 90%, compared to the natural $\hbox {Ca}^{2+}$ signalling.

## Discussion

Our study has found that $\hbox {Ca}^{2+}$ fluctuations are the main source of noise in the astrocyte-based logic gates, as observed in both the wet lab experiments (Fig. 3b,c) and simulations (Fig. 4b). These fluctuations are caused by both the $\hbox {Ca}^{2+}$ intra and intercellular signalling. In the case of intercellular signalling, the noise is dependent on the topology of the astrocyte cell population as $\hbox {Ca}^{2+}$ ions can randomly propagate between the cells in the population. We also know from multiscale analysis^30^ that even single-cell irregularities can result in random fluctuations of $\hbox {Ca}^{2+}$ propagation. These noise and random fluctuations can result in unreliable logic operation. Moreover, as shown in both Fig. 3b and 4a,e, the relationship between the threshold as well as the $T_b$ can lead to false results in the logic gate computation. Accuracy can go up to above 90% levels when $T_b$ is higher than 30s for the AND gate, and for 25s in the OR gate. This is because a fluctuation is shown to decrease when increasing the $T_b$ as shown in Fig. 4b,f. However, as shown in Fig. 4c,d, the decision of a threshold is dependent on the impact of the output accuracy, false positive and false negatives results, topology structure, the position where the logic gates are placed as well as the system dynamics. This can be improved with the usage of fuzzy systems to calculate adaptable time-varying computing thresholds. The usage of a reinforced-learning approach to decide parameters such as the threshold and $T_b$ can lead the system to optimum results when information about the network topology is not available. This is where the benefits of the reinforced-learning platform manifest: it determines the optimum $T_b$, by analyzing the propagation noise that is transmitted through the molecular communication simulator until it converges to a value resulting in the least amount of noise, irrespective of the topology. An analysis of the computational capacity of this system could explain what are the upper limits of signal processing by eukaryote cells. Our experiments in Fig. 3 shows that different compounds amplify the $\hbox {Ca}^{2+}$ signalling for the output of the logic gate, allowing us to set different thresholds for the AND and OR gates. This also means that the topology of the input links plays a role in ensuring that the optimum $\hbox {Ca}^{2+}$ signals should flow into the output population in order to obtain accurate results from the logic computation. The results from the experiments that used the compounds for elevating the $\hbox {Ca}^{2+}$ signals of the input links was used in the simulations to determine the impact of $\hbox {Ca}^{2+}$ ion concentration propagation on the static delay analysis of the logic operation (Fig. 5). The simulation has shown that higher concentration of $\hbox {Ca}^{2+}$ propagated between the cells, leads to faster diffusion, which lowers the delay that can lead to higher iterative numbers of logic computations. Therefore, a design for neural–molecular computing chips could include substrates with the compound mixed with the engineered astrocyte culture, to further amplify the $\hbox {Ca}^{2+}$ ion production as well as propagation.

The majority of biomolecular computing techniques^10,31,32^ developed to date rely on the DNA transcription and translation processes, which limits their operation for future in-vivo applications. Such approaches require insertion of complex genetic circuits into the cells that can result in gene expressions that can be damaging to their biological environment, possibly affecting tissue homoeostasis^33^. While there are benefits from the use of cell-free expression techniques, where the machinery are not required to be embedded in a living cell, the operation can be unreliable when all components are required to work together within a liquid environment. Our approach can partially eliminate these issues by 1) embedding simpler synthetic genes into the cell’s genome, and 2) providing a new approach for brain bio-electronics that utilizes engineered astrocytes, where the engineering is only based on manipulating the flow of $\hbox {Ca}^{2+}$ ions (using the compounds) and thresholds to achieve gate behaviours. An important benefit of using astrocytes for neural–molecular computing is the ease of integration into the brain tissue, where they can easily connect with natural neurons in order to receive incoming signals as well as produce output signals. Our work lays the foundation for neural–molecular computing chips that can embed logic circuits built from gates of engineered astrocytes. Therefore, future work will need to investigate how the astrocyte-based logic gates can be connected into a circuit^34^. The neural–molecular computing on a chip that houses the engineered astrocytes can be designed and constructed from biocompatible material, avoiding the need for silicon technology to perform computation.

## Conclusion

The vision of molecular computing is to perform unconventional computing using biological systems, and in particular through the interaction of molecules produced by cell machinery. Over the years, many molecular computing approaches have been developed, using DNA, where computing functions is achieved through multiple DNA molecules interacting, as well as using cells, such as bacteria. In this paper, we take an alternative approach where molecular computing is achieved through the engineering of Eukaryotic cells, and in particular, astrocytes. By engineering the threshold of $\hbox {Ca}^{2+}$ ions that flow between the cells, AND and OR gates can be developed. The paper first demonstrated through wet lab experiments AND and OR gates that can be developed using hGPR-17 synthetic gene expression, with incoming $\hbox {Ca}^{2+}$ signals simulated from chemical compounds (*MDL*29, 951 and *T*0510.3657) added to the culture. The results showed that AND and OR gate behaviour can be achieved, provided that the threshold is set accordingly, and this threshold will be determined by the quantity of chemical compound added to the culture. The paper also presented a reinforced learning platform for logic gate design that is agnostic to any cell culture and can be used to determine the optimum $\hbox {Ca}^{2+}$ activated level and input transmission period $T_b$. The validation was performed using a $\hbox {Ca}^{2+}$-signalling based molecular communication simulator. The simulations showed that for any type of input topology of astrocyte network, there is an optimum value for the $\hbox {Ca}^{2+}$ activated level and input transmission period $T_b$, and this was validated through the reinforced learning platform. Future work can use the reinforced learning platform to design the timing of the input signals as well as the activated level for any type of cell culture. The work presented in this paper lays the foundation for future neural–molecular computing on a chip that is constructed from biological cells that perform computing functions, minimizing the need for silicon technology. This, in turn, can result in future brain implants that are controlled and operated via molecular computing logic circuits.

## Supplementary information

Supplementary material 1
