# Supplementary material for: Engineering calcium signaling of astrocytes for neural–molecular computing logic gates
Source: Sci Rep. 2021 Jan 12;11:595. doi: 10.1038/s41598-020-79891-x (PMC7803753; doi:10.1038/s41598-020-79891-x)
Supplement: Supplementary file 1 — Supplementary material 1 [file 41598_2020_79891_MOESM1_ESM.pdf]

Supplementary Information for "Engineering Calcium Signaling of Astrocytes for Neural-Molecular Computing Logic Gates"

Michael Taynnan Barros[1,2,*], **Phuong Doan**[2], **Meenakshisundaram Kandhavelu**[2], **Brendan Jennings**[3], **and Sasitharan Balasubramaniam**[3]

[1]School of Computer Science and Electronic Engineering, University of Essex, Colchester, UK.
[2]BioMediTech, Faculty of Medicine and Health Technology, Tampere University, P.O.Box 553, 33101 Tampere, Finland.
[3]Telecommunication Software & Systems Group (TSSG), Waterford Institute of Technology (WIT), Ireland
[*]m.barros@essex.ac.uk

ABSTRACT

Here we present the mathematical framework used in the paper. The code of the simulations described here can be found in https://github.com/michaelbarros/astrocytes_population_sim.

Computational model of astrocytes intracellular signalling

In this section we introduce a model describing Ca^{2+} oscillations in astrocytes that was proposed by Lavrentovich and Hemkin[1]. The model is in accordance with experimental observation[1]. We have Ca^{2+} pool storage models, which includes: Ca^{2+} concentration in the cytosol (C_a) (Eq. 1); Ca^{2+} concentration in the endoplasmic reticulum (E_a) (Eq. 2); and IP_3 concentration (I_a) (Eq. 3). They are represented by the following equations:

$$\frac{dC_a}{dt} = \sigma_0 - \kappa_o C_a + \sigma_1 - \sigma_2 + \kappa_f (E_a - C_a) + \sigma_4 \tag{1}$$

$$\frac{dE_a}{dt} = \sigma_2 - \sigma_1 - \kappa_f (E_a - C_a) \tag{2}$$

$$\frac{dI_a}{dt} = \sigma_3 - \kappa_d I_a \tag{3}$$

where σ_0 is the flow of Ca^{2+} from the extracellular space into the cytosol [1], $\kappa_o C_a$ is the rate of Ca^{2+} efflux from the cytosol to the extracellular space, $\kappa_f(E_a - C_a)$ is the leak flux from the endoplasmic reticulum into the cytosol and $\kappa_d I_a$ is the degradation of IP_3.

The σ_1 term (Eq. 4), models the Ca^{2+} flux from the endoplasmic reticulum to the cytosol via IP_3 stimulation. In common with excitable and non-excitable cells, this mechanism directly affects the cytosolic concentration of Ca^{2+}. It is represented as:

$$\begin{aligned} \sigma_1 &= 4\Sigma_{M3} \frac{\kappa_{C1}^n C_a^n}{(C_a^n + \kappa_{C1}^n)(C_a^n + \kappa_{C2}^n)} \\ &\quad \cdot \frac{I_a^m}{\kappa_I^m + I_a^m} (E_a - C_a) \end{aligned} \tag{4}$$

where Σ_{m3} is the maximum flux value of Ca^{2+} into the cytosol, κ_{C1}^n and κ_{C2}^n are the activating and inhibiting variables for the IP_3 and the m and n are the Hill coefficients.

The efflux of Ca^{2+} from the sarco(endo)plasmic reticulum to the endoplasmic reticulum is modelled as σ_2:

$$\sigma_2 = \Sigma_{M2} \frac{C_a^2}{\kappa_2^2 + C_a^2} \tag{5}$$

[1]This term can be extended into a voltage dependent term, called voltage-gated Ca^{2+} channels, which was further studied in[2].

where Σ_{M2} is the maximum flux of Ca^{2+} in this process. Finally, σ_3 describes IP_3 generation by the Phosphoinositide phospholipase C (PLC) protein:

$$\sigma_3 = \Sigma_p \frac{C_a^2}{\kappa_p^2 + C_a^2} \tag{6}$$

where Σ_p is the maximum flux of Ca^{2+} in this process, and p is the Hill coefficient.

The σ_4 is the external Ca^{2+} influx used to model the direct amplification of calcium based on the addition of MDL29,951, T 0510.3657 and GPR17. We use a Piecewise linear regression model from the experiments showing in Fig 3.b).

Gap Junctions Model

A stochastic model of gap junction behaviour was introduced by Baigent et al.[3] and first studied for molecular communication by Kilinc and Akan[4]. The model considers voltage-sensitive gap junctions which are assumed to have two states of conductance for each connexin: an open state with high conductance and a closed state with low conductance. Based on this, we consider four basic combinations of states from each connexin of the connexon:

- *State HH*: Both gates are in a high conductance state. This probability is denoted by p_{HH};
- *State HL*: One gate is in a high conductance state, and the other is in a low conductance state. This probability is denoted by p_{HL};
- *State LH*: One gate is in a low conductance state, and the other is in a high conductance state. This probability is denoted by p_{LH};
- *State LL*: Both gates are in a low conductance state. This probability is denoted by p_{LL}.

Experimental validation of the model indicated that the LL state appears to present very low occurrence rates[5], thus we neglect that state here. Thus, the probabilities should follow:

$$p_{HH} + p_{HL} + p_{LH} = 1 \tag{7}$$

Moreover, p_{HH}, p_{HL} and p_{LH} are interrelated as follows:

$$\frac{dp_{HL}}{dt} = \beta_1(\vartheta_j) \times p_{HH} - \alpha_1(\vartheta_j) \times p_{LH} \tag{8}$$

$$\frac{dp_{LH}}{dt} = \beta_2(\vartheta_j) \times p_{HH} - \alpha_2(\vartheta_j) \times p_{HL} \tag{9}$$

where the control of the gap junctions permeability is mediated through the potential difference of the membrane of two adjacent cells (ϑ_j), the gate opening rate is α and gate closing rate is β. The terms $\alpha_1(\vartheta_j)$, $\alpha_2(\vartheta_j)$, $\beta_1(\vartheta_j)$ and $\beta_2(\vartheta_j)$ are defined as:

$$\alpha_1(\vartheta_j) = \lambda e^{-A_\alpha(\vartheta_j - \vartheta_0)} \tag{10}$$

$$\alpha_2(\vartheta_j) = \lambda e^{A_\alpha(\vartheta_j + \vartheta_0)} \tag{11}$$

$$\beta_1(\vartheta_j) = \lambda e^{A_\beta(\vartheta_j - \vartheta_0)} \tag{12}$$

$$\beta_2(\vartheta_j) = \lambda e^{-A_\beta(\vartheta_j + \vartheta_0)} \tag{13}$$

where ϑ_0 is the junctional voltage at which the opening and closing rates of the gap junctions have the same common value λ, and A_α and A_β are constants that indicate the sensitivity of a gap junction to the junctional voltage.

Simulation

We consider a cellular tissue space (S) composed of $I \times J \times K$ cells (c), where $c_{i,j,k}$ ($i = 1 \ldots I$; $j = 1, \ldots J$ and $k = 1, \ldots K$) denotes an arbitrary cell in the tissue. The cells are connected with a maximum of six neighbouring cells. In the case of the excitable and non-excitable cells, the organisation of the cells is assumed to be a layered lattice. However, for astrocytes, the organisation is going to depend on the type of topology connection. We use a simple regular connection to perfectly match our lattice model, that is based on the study of astrocytes topologies[9].

Table 1. Experimental variable values for Cx43 of astrocytes[3678].

Variable	Value
λ	0.37
ϑ_j mV	90
ϑ_0 mV	60
A_α $(\text{mV})^{-1}$	0.008
A_β $(\text{mV})^{-1}$	0.67

Consider that each cell contains a set of internal reactions of P1 and P2 pools. Each reaction and pool for a specific cell type were defined in the previous section. The stochastic solver computes the values of each pool over time, selecting and executing scheduled reactions. The pool will be negatively or positively affected by a constant α when a specific reaction is executed.

Modelling diffusion in a cellular tissue area captures the temporal-spatial dynamics of intercellular Ca^{2+} signaling. We use Ca^{2+} concentration difference to model this temporal-spatial characteristic, as follows[10]:

$$Z_\Delta(i,j,k,n,m,l) = \frac{D}{v}(|Z_{n,m,l} - Z_{i,j,k}|) \times p_{(.)} \tag{14}$$

where $n \in (i-1, i+1)$, $m \in (j-1, j+1)$, $l \in (k-1, k+1)$, D is the diffusion coefficient, v is the volume of the cell, and Z_Δ is the difference in Ca^{2+} concentration between the cells. $p_{(.)}$ is the probability of the gap junction opening and closing. Based on the gap junction probabilities, we define three different diffusion reactions for each cellular connection. Such reactions are the multiplication of the probabilities (p_{HH}, p_{HL} and p_{LH}) with the regular cell-to-cell diffusion probability.

Stochastic solver

We present in this section a stochastic solver, which determines the quantity of each pool over time. At each time step, the Gillespie algorithm[11] is executed to select a random cell and a random internal reaction of that cell, also scheduling a time step (t) to each one of them.

The process of executing one of the distinct reactions in R requires a scheduling process divided in two phases—selecting a reaction and selecting a time step. Each reaction is allocated a reaction constant (a_r). Considering that α_0 is the summation of all a_r in R, the next reaction chosen r_u will be:

$$r_u = \text{MAX}\left\{ \frac{a_{r_j}}{\alpha_0} = \frac{a_{r_j}}{\sum_{j=1}^{|R|} a_{r_j}} \right\}, u \in \mathbb{N}, u \in R \tag{15}$$

which follows the *roulette wheel selection* process, which selects the events based on their probability values. However, u must satisfy the following restriction:

$$\sum_{j=1}^{u-1} \frac{\alpha_{r_j}}{\alpha_0} < \rho_2 \leq \sum_{j=1}^{u} \frac{\alpha_{r_j}}{\alpha_0} \tag{16}$$

in which ρ_2 is a uniform random variable with values in the range $(0,1)$.

At each time step (t), a time lapse (τ_t) is derived based on α_0, and is represented as:

$$\alpha_0 \cdot \tau_t = \ln \frac{1}{\rho_1} \tag{17}$$

in which ρ_1 is a uniform random variable with values in the range $(0,1)$. This process ends when $\sum_{i=0}^{|T|} \tau_i < t_\theta$, where T is the set of t and t_θ is the maximum simulation time.

Logic gate model

The synthetic logic gates programming is made on the reaction-diffusion process that governs the Ca^{2+} signalling-based molecular communications model. We basically analyse the molecular concentration of the cell in order to defined the Ca^{2+}

Table 2. Simulation parameters for astrocytes[1].

Variable	Value
C_a	$0.1\mu M$
E_a	$1.5\mu M$
I_a	$0.1\mu M$
σ_0	$0.05\ \mu M$
κ_o	$0.5\ \mathrm{s}^{-1}$
κ_f	$0.5\ \mathrm{s}^{-1}$
κ_d	$0.08\ \mathrm{s}^{-1}$
Σ_{M2}	$15\mu M/s$
κ_2	$0.1\mu M$
Σ_p	$0.05\mu M/s$
κ_p	$0.3\mu M$
n	2.02
κ_{C1}	$0.15\mu M$
κ_{C2}	$0.15\mu M$
κ_I	$0.1\mu M$
Σ_{M3}	$40.0\mathrm{s}^{-1}$
m	2.2
D	$350\mu m^2/s$

concentration threshold that triggers the output of the logic gate. This approach is inspired by[12,13], where a recurrent biophysical signalling pathway model was used for the logic gate design. The stages for logic gate operation are as follows: First, the Ca^{2+} concentration threshold is defined for a specific logic operation function followed by the synthetic gene implementation. Secondly, the upcoming Ca^{2+} from neighbouring cells through intercellular signalling are considered as inputs. In our case, we have two inputs from two different cells. All inputs are being transmitted to the logic gate cell during the *signalling period* (T_b). The inputs interfere with the concentration in the cell cytosol $[C_a]$ alongside with the existing intracellular Ca^{2+} signalling. Logic gates have two known states, an "OFF" state or "0" and an "ON" state or "1". The transition between states is performed when $[C_a] > Ca^{2+}$ concentration threshold for the "ON" state and $[C_a] \leq Ca^{2+}$ concentration threshold for the "OFF" state.

Static Timing Analysis

Our analysis on the delay is based on conventional digital circuit static timing analysis. Based on a value of delay that refers to the time a logic gate gives a complete output, d, and a number of operations that is established, n, our total delay of operation time D is the linear progression of the given initial delay value, which equals to

$$D = \sum_{0}^{n} d = d * n \tag{18}$$

References

1. Lavrentovich, M. & Hemkin, S. A mathematical model of spontaneous calcium(ii) oscillations in astrocytes. *Journal of Theoretical Biology* **251**, 553–560 (2008).
2. Zeng, S., Li, B., Zend, S. & Chen, S. Simulation of spontaneous ca2+ oscillations in astrocytes mediated by voltage-gated calcium channels. *Biophysical Journal* **97**, 2429–2437 (2009).
3. Baigent, S., Stark, J. & Warner, A. Modelling the effect of gap junction nonlinearities in systems of coupled cells. *Journal of Theoretical Biology* **186**, 223–239 (1997).
4. Kilinc, D. & Akan, O. B. An information theoretical analysis of nanoscale molecular gap junction communication channel between cardiomyocytes. *IEEE Transactions on Nanotechnology* **12**, 129–136 (2013).
5. Bukaukas, F. F., Bukauskiene, A., Bennett, M. V. L. & Verselis, V. K. Gating properties of gap junction channels assembled from connexin43 and connexin 43 fused with green fluorescent protein. *Biophysics Journal* **81**, 137–152 (2013).

6. Viliunas, V., Weingart, R. & Brink, P. R. Formation of heterotypic gap junction channels by connexins 40 and 43. *Cirulation Research* **86**, E42–E49 (2000).

7. Moreno, A. P., Laing, J. G., Beyer, E. C. & Spray, D. C. Properties of gap junction channels formed of connexin 45 endogenously expressed in human hepatoma (skhep1) cells. *The American Journal of Physiology* **268**, 356–365 (2000).

8. Viliunas, V. Biophysical properties of connexin-45 gap junction hemichannels studied in vertebrate cells. *The journal of general physiology* **119**, 147–164 (2002).

9. Lallouette, J., Pitta, M. D., Ben-Jacob, E. & Berry, H. Sparse short-distance connection enhace calcium wave propagation in a 3d model of astrocytes networks. *Frontiers in Computation Neuroscience* **8**, 1–18 (2014).

10. Nakano, T. & Liu, J.-Q. Design and analysis of molecular relay channels: An information theoretic approach. *IEEE Transactions on NanoBioscience* **9**, 213–221 (2010).

11. Gillespie, D. T. Exact stochastic simulation of coupled chemical reactions. *Journal of Physical Chemistry* **81**, 2340–2361 (1977).

12. Stetter, M., Schurmann, B. & Hofstetter, M. Logical nano-computation in enzymatic reaction networks. In *2006 1st Bio-Inspired Models of Network, Information and Computing Systems*, 1–7 (2006).

13. Hiratsuka, M., Aoki, T. & Higuchi, T. Enzyme transistor circuits for reaction-diffusion computing. *IEEE Transactions on Circuits and Systems I: Fundamental Theory and Applications* **46**, 294–303 (1999).
